# Assessment of neonatal BPBP

**DOI:** 10.1186/1753-6561-9-S3-A15

**Published:** 2015-05-19

**Authors:** Michael Tonkin

**Affiliations:** 1 Department of Hand Surgery, University of Sydney, New South Wales, 2006 Australia

## 

The incidence of neonatal brachial plexus palsy is about 1 in 1000 live births with up to 20% having some sequelae but only 8% requiring surgery. Surgical complications can occur and therefore the indications for and the timing of surgery is of paramount importance. The indications are determined by clinical examinations and investigations. It is necessary to rule out differential diagnoses but we do need to consider whether investigations that we perform accurately reveal the extent and severity of the neurological lesion. These investigations are x-rays, CT myelography, MRI and electromyography and nerve conduction studies. None of these investigations would appear to be as reliable as they are when they are applied to adults. CT myelography and an MRI require general anaesthetic . There is a low sensitivity but a high specificity of root avulsion in the presence of a pseudomeningocele and absence of rootlets. Improving fast spin-echo MRI techniques may be of greater benefit in the future.

The reliability of electromyography and nerve conduction studies differs from that in adult lesions which may be due to a number of different factors. They are accurate for neuropraxia and avulsion but less satisfactory for a neuroma in continuity, in determining recovery of function and indeed in their performance. The results of EMG and NCS may be unduly optimistic.

Therefore, clinical examination remains the main basis upon which surgery is indicated. The thesis of Tassin and the work of Gilbert have suggested that the presence or absence of elbow flexion is a good indicator of the return of shoulder function. The indications for surgery in C5-6 and C5-6-7 lesions are based upon this clinical finding. Others have questioned as to whether this is an accurate method of determination of surgical necessity. Clarke claims improvement of the predictability of recovery by including the return of other muscle functions and combining these into a test score, but it is uncertain whether this score improves the accuracy of assessment of C5-6 lesions. Most would agree that surgery by 3 months in pan-plexus lesions is appropriate.

I perform routine clinical examinations, preferably on a 2 week basis from the time of the first presentation. Close documentation from as early an age as possible is vital to determining the presence or absence of specific muscle function and the rate of any recovery. I operate for failure of biceps recovery at 4 months in C5,6+/-7 lesions and by 3 months in pan-plexus lesions. Beyond this time, neurosurgical exploration may be indicated for failure to improve. Nerve transfers, for instance accessory to suprascapular nerve, have been effective up to 15 months of age. X-rays of the chest, cervical spine and shoulder are routine and, although the reliability of EMG and NCS is less than satisfactory, these are performed at 3 months. MRI examination will become a routine investigation but at the moment it is not my practice to routinely order these on all children.

